# The PUF Protein Family: Overview on PUF RNA Targets, Biological Functions, and Post Transcriptional Regulation

**DOI:** 10.3390/ijms19020410

**Published:** 2018-01-30

**Authors:** Ming Wang, Laurent Ogé, Maria-Dolores Perez-Garcia, Latifa Hamama, Soulaiman Sakr

**Affiliations:** IRHS, Agrocampus-Ouest, INRA, Université d’Angers, SFR 4207 QUASAV, F-49045 Angers, France; Ming.Wang@agrocampus-ouest.fr (M.W.); laurent.Oge@agrocampus-ouest.fr (L.O.); maria-dolores.perez-garcia@agrocampus-ouest.fr (M.-D.P.-G.); latifa.hamama@agrocampus-ouest.fr (L.H.)

**Keywords:** PUF protein, post-transcriptional, regulation, plant, RNA-binding motifs

## Abstract

Post-transcriptional regulation of gene expression plays a crucial role in many processes. In cells, it is mediated by diverse RNA-binding proteins. These proteins can influence mRNA stability, translation, and localization. The PUF protein family (Pumilio and FBF) is composed of RNA-binding proteins highly conserved among most eukaryotic organisms. Previous investigations indicated that they could be involved in many processes by binding corresponding motifs in the 3′UTR or by interacting with other proteins. To date, most of the investigations on PUF proteins have been focused on *Caenorhabditis elegans*, *Drosophila melanogaster*, and *Saccharomyces cerevisiae*, while only a few have been conducted on *Arabidopsis thaliana*. The present article provides an overview of the PUF protein family. It addresses their RNA-binding motifs, biological functions, and post-transcriptional control mechanisms in *Caenorhabditis elegans*, *Drosophila melanogaster*, *Saccharomyces cerevisiae*, and *Arabidopsis thaliana*. These items of knowledge open onto new investigations into the relevance of PUF proteins in specific plant developmental processes.

## 1. Introduction

In most eukaryotic organisms, gene expression is commonly regulated at the transcriptional and posttranscriptional levels; this is considered as a powerful strategy for these organisms to flexibly adapt their growth and development to environmental inputs. Extensive investigations have reported that RNA-binding proteins (RBPs) regulate many aspects of RNA processing, such as RNA splicing, polyadenylation, capping, modification, transport, localization, translation, and stability, called RNA metabolism [1,2,3,4,5]. The resolution of protein structures and the functional characterization of RBPs have shown that these proteins possess several conserved motifs and domains such as RNA-recognition motifs (RRMs), zinc fingers, K homology (KH) domains, DEAD/DEAH boxes (highly conserved motif (Asp-Glu-Ala-Asp) in RNA helicases), Pumilio/FBF (*Caenorhabditis elegans* Pumilio-*fem-3* binding factor, PUF) domains, and pentatricopeptide-repeat (PPR) domains [6]. 

The Pumilio RNA-binding protein family—the PUF family—is a large family of RBPs found in all eukaryotes; the number of PUF gene copies in each model organism is highly variable. The PUF family is mainly involved in post-transcriptional control by binding to specific regulatory *cis*-elements of their mRNA targets. Through this interaction they govern RNA decay and translational repression [7]. They also act by promoting ribosome stalling and facilitating the recruitment of microRNAs (miRNAs) and chromosomal instability [8,9,10,11]. Therefore, PUF protein influences the expression level of their target gene dramatically through the post-transcriptional level. For example, Puf6p can inhibit Asymmetric Synthesis of HO (*ASH1*) mRNA translation in yeast. The experiment from Gu et al. showed that the *ASH1* in *puf6* mutant had a higher expression level than in the wild type [12]. Suh et al. indicated that FBF, a PUF protein in *Caenorhabditis elegans*, can represses *gld-1* expression through interact with *gld-1* mRNA. GLD-1 level increased approximately sixfold in *fbf* mutant than in wild type [13].

The present article provides a rapid overview of PUF proteins, especially their binding motifs, biological functions, and regulation mechanisms, which seem to be conserved among eukaryotes. Given that our knowledge of the functional roles of RBPs in plants is lagging far behind our understanding of their roles in other organisms, this article ends by briefly underlying the interest of investigating the role of the PUF family in certain key mechanisms of plant functioning. 

## 2. RNA-Binding Target of PUF Proteins 

**Figure 1:**
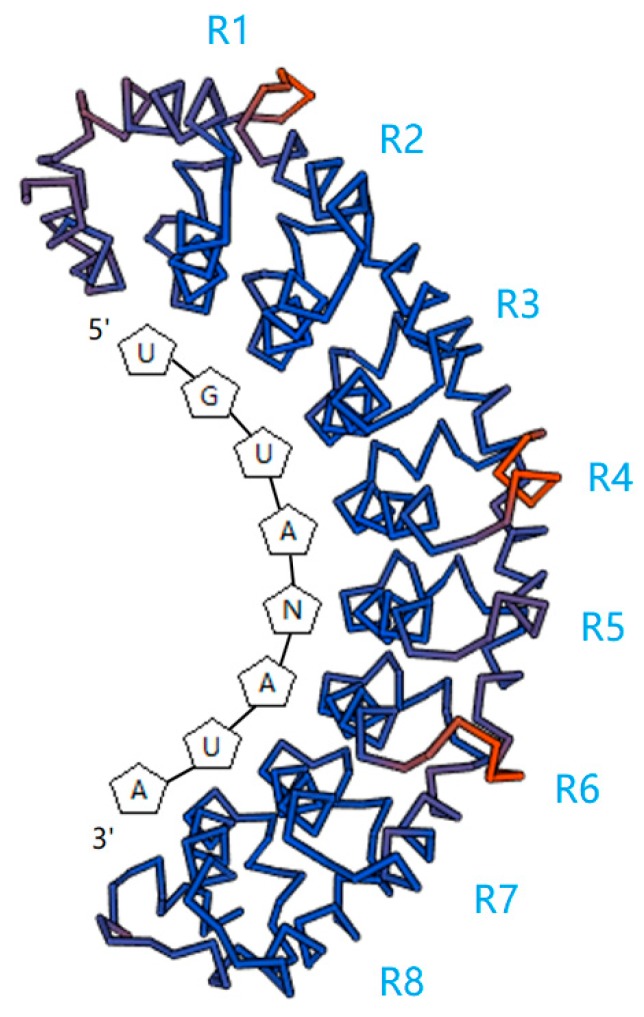
Predictive stick models of the APUM2 (*Arabidopsis thaliana* Pumilio 2, AT2G29190) PUM-HD (Pumilio homology domain) bound to its target motif. The analysis result based on the research of Francischini and Quaggio [42]. They showed that the PUM-HD of APUM2 bound to the core nucleotides of 5′-UGUANAUA-3′. The each repeat of PUM-HD bound to corresponding nucleotide through Van der Waals force. The protein structure was generated by SWISS-MODEL (https://swissmodel.expasy.org/) [44,45,46,47].

*Drosophila melanogaster* Pumilio (DmPUM) and *Caenorhabditis elegans* Pumilio-*fem-3* binding factor (FBF) are the two founding members of the PUF protein family [14]. These canonical PUF proteins contain an extensively conserved RNA-binding domain (the Pumilio homology domain, PUM-HD), composed of eight consecutive α-helical PUF repeats that adopt a crescent-shaped structure [1,14,15,16,17,18]. The crystal structure of PUM-HD revealed that each of the eight PUF repeats specifically recognizes a single nucleotide in its target RNA, and can thereby bind to as many as eight consecutive nucleotides, and this binding model is conserved [7,19]. PUF proteins initially appeared to bind RNAs containing a 5′-UGU-3′ triplet (Figure 1), and were thought to act cooperatively with other proteins [14,19,20,21,22,23,24]. For instance, DmPUM binds to the Nanos response element (NRE) that harbors motifs A (5′-GUUGU-3′) and B (5′-AUUGUA-3′) in the 3′UTR of *hunchback* (*hb*) mRNA [25,26,27]. Each motif contains the core UGU triplet and interacts with one Pumilio protein in a cooperative manner [28]. In *Caenorhabditis elegans*, FBF-1 and FBF-2 (*C. elegans fem-3 mRNA-binding factors 1 and 2*, two nearly identical proteins collectively called FBF) bind to the same core RNA-binding sites that possess the UGU trinucleotide and an AU pair located 3 nucleotides downstream (5′-UGUDHHAUA-3′; D, A or G or T; and H, A or C or T) [29]. The binding activity of FBF-2 and other *C. elegans* PUMs (PUF6 and PUF11) is enhanced by an additional binding pocket for cysteine located upstream [30]. In *Saccharomyces cerevisiae*, Puf3p, which localized in mitochondria, binds the RNA sequence 5′-UGUANAUA-3′, while yeast Puf4p and Puf5p recognize 5′-UGUR-3′ (R, purine)-containing sites [31,32]. The experiment also indicated that yeast Puf4p and Puf5p mainly function in nucleolus [33]. To be functional, PUF1p (Jsn1p) and the closely related protein PUF2p bind RNAs containing 5′-UAAU-3′ rather than the more common motif 5′-UGUR-3′. This difference is assigned to their “non-canonical” features consisting of fewer PUF-repeats [34]. In murine, PUM2, which contains a C-terminal RNA-binding domain related to the *Drosophila Pumilio* homology domain (PUM-HD), can bind to the consensus sequence 5′-UGUANAUARNNNNBBBBSCCS-3′ (N, any base; R, A or G; B, C or G or T; and S, G or C) [35]. According to many authors [7,19,36], the binding model of each PUF repeat to an RNA base could be similar. However, PUF proteins can recognize RNA sequences beyond the PUM-HD scaffold and also interact with non-cognate sequences, underlying the higher complexity and adaptability of their binding activity [37,38,39]. To support this point, other studies showed that PUF proteins bind to CDSs or 5′UTRs. They bind to *paralytic* (*para*) in the CDS region of its mRNA, which encodes the Drosophila voltage-gated sodium channel paralytic [40]. In *Cryptococcus neoformans*, Pum1, an ortholog of both *S. cerevisiae* Puf3p and *Drosophila melanogaster* Pumilio, can only bind to the consensus binding element 5′-UGUACAUA-3′ in the 5′UTR of its own mRNA to participate to the regulation of hyphal morphogenesis [41]. 

In plants, few investigations have been led to discover PUF-binding sites and thereby their role in plant growth and development. The experimental results from Tam et al. indicated that AtPum2, an Arabidopsis PUF protein, binds the RNA of *Drosophila* Nanos Response Element I (NRE1) 5′-UGUAUAUA-3′ located in its 3′UTR [7]. They also showed that APUM1 to APUM22 can shuttle between nucleus and cytoplasm through the exportin1 mediated pathway. However, APUM23 and APUM24 localized in nucleus [7]. Through three-hybrid assays, Francischini and Quaggio showed that among the 25 PUF members identified in Arabidopsis, APUM1 to APUM6 can specifically bind to the Nanos response element sequence, which is also recognized by Drosophila Pumilio proteins [42]. They also identified an APUM-binding consensus sequence through three-hybrid screening assay in Arabidopsis RNA library, i.e., a 5′-UGUR-3′ tetranucleotide sequence reported to be present in all targets of the PUF family [1]. However, the “non-canonical” Arabidopsis PUM23 (APUM23) binding sequence is 10 nucleotides long, contains a 5′-UUGA-3′ core sequence, and has a preferred cytosine at nucleotide position 8 [43]. These investigations showed that the consensus PUF-binding motif may be ubiquitous among eukaryotes, but no study in plants has reported a PUF motif in other regions than the 3′UTR. 

## 3. Putative Biological Functions of PUF Proteins 

Many studies demonstrated that individual PUF proteins can recognize hundreds of unique transcripts, suggesting that this family of proteins can regulate many aspects of eukaryote mechanisms, including stem cell control, developmental patterning, neuron functioning, and organelle biogenesis (Table 1). Up to now, the most extensive investigations about PUF proteins have focused on *Caenorhabditis elegans*, *Drosophila melanogaster*, and *Saccharomyces cerevisiae*, and only few reports are available about plants.

Based on the biological functions analyzed so far, PUF proteins significantly control diverse processes in these species. In Drosophila, Pumilio was identified initially from its requirement for embryonic development through regulating *Hunchback* (an important morphogen gene), in collaboration with the zinc finger protein Nanos [70]. Other processes such as stem cell proliferation, motor neuron function, and memory formation are under the control of Pumilio [70]. In *Caenorhabditis elegans*, FBFs control gametogenesis by mediating the sperm/oocyte switch, while PUF8 displays several functions, including the sperm-oocyte switch during normal development and its antagonistic effects on germline stem cell proliferation [14]. The latter depends on the genetic context, in that PUF8 and MEX3 (a KH-type RNA-binding protein) redundantly promote germline stem cell proliferation in *Caenorhabditis elegans* [52]. PUF8 also acts as a repressor of germline stem cell proliferation in temperature-sensitive *glp-1*(*ar202*) gain-of-function mutants whose GLP-1 activity is high [71]. Two groups of PUF/RNA-binding proteins, PUF-3/11 and PUF-5/6/7, play different roles in *Caenorhabditis elegans* oogenesis. All of them are involved in oocyte formation, but PUF-3/11 limits oocyte growth while PUF-5/6/7 promotes oocyte organization and formation [72]. Salvetti et al. identified a homologous protein of *Drosophila Pumilio* in *Dugesia japonica*, named *DjPum*. It is expressed in planarian stem cells and involved in the formation of the regenerative blastema [73]. Moreover, these same authors showed that *DjPum* is essential for neoblast maintenance. 

The yeast PUF protein Mpt5p regulates the stability of *HO* mRNA by stimulating removal of its poly(A) tail [74]. *HO* is involved in mating-type switching in yeast: it introduces double-stranded DNA breaks that initiate recombination [75]. The PUF3 protein plays a key role because it can bind and regulate more than 100 mRNAs that encode proteins with mitochondrial functions [76]. A bioinformatics method showed that *hmt1*, a protein arginine *N*-methyltransferase, and *dut1*, which encodes a dUTP pyro-phosphatase, were predicted as putative mRNA targets of PUF4p in yeast [33]. PUF5p is a broad RNA regulator in *S. cerevisiae* that binds to more than 1000 RNA targets; it makes up around 16% of the yeast transcriptome. These RNAs regulate many aspects of *S. cerevisiae* development such as embryonic cell cycle, cell wall integrity, or chromatin structure [77]. Nop9, an *S. cerevisiae* PUF protein, recognizes sequences and structural features of 20S pre-rRNA near the nuclease cleavage site. It also associates with the SSU processome/90S pre-ribosome through protein–protein interactions before its 20S pre-rRNA target site is transcribed [78]. Mpt5p (also called Puf5p or Uth4p) promotes temperature tolerance and increased replicative life span in *S. cerevisiae* through an unknown mechanism thought to be partly involved in the cell wall integrity (CWI) pathway. mpt5Δ mutants also have a short life span; this defect is suppressed when CWI signaling is activated [79]. 

Certain reports reveal the pathways in which PUF proteins are involved in other species. *Peronophythora litchi PIM90* encodes a putative PUF protein; its expression is relatively lower during cyst germination and plant infection, but it is highly expressed during asexual and sexual development [80]. In *Plasmodium falciparum*, *PfPUF1* plays an important role in the differentiation and maintenance of gametocytes, especially female gametocytes [81]. In the *PfPUF1*-disrupted lines, gametocytes appeared normal before stage III but subsequently exhibited a sharp decline in gametocytemia. In *Cryptococcus*, *Pum1* is auto-repressive during growth, controls its own morphotype expression, and positively stabilizes the expression of *ZNF2* (a filamentation regulator) to achieve the filamentous morphotype required for sexual development [41]. In humans, the two Pumilio proteins PUM1 and PUM2 were identified as positive regulators of Retinoic acid-inducible gene I (RIG-I) signaling, which plays a pivotal role in innate immunity [82]. Overexpression of PUM1 and PUM2 increased IFN-β (an important factor in RIG-I signaling) promoter activity induced by Newcastle disease virus (NDV), while the opposite effect was reported when these Pum proteins were knocked down [82]. 

PUF proteins may also act as post-transcriptional repressors through a conserved mechanism in Plant. APUM5 is associated with both biotic and abiotic stress responses [66]. APUM5-overexpressing plants showed hypersensitive phenotypes under salt and drought treatment during germination at the seedling stage and vegetative stage. Further results indicated that the APUM5-Pumilio homology domain (PHD) protein bound to the 3′UTR of many salt and drought stress-responsive genes containing putative Pumilio RNA-binding motifs in their 3′UTR [66]. AtPUM23 regulates leaf morphogenesis by regulating the expression of KANADI (*KAN*) genes. KANADI genes are members of the GARP family, key regulators of abaxial identity [68]. Moreover, PUF proteins have also been predicted to participate in many mechanisms in Arabidopsis, such as responses to nutrients, light, iron deficiency, ABA (abscisic acid) signaling, and osmotic stress [7]. For example, APUM23, a nucleolar constitutively PUF-domain protein expressed at higher levels in metabolically active tissues, was upregulated in the presence of glucose or sucrose. APUM23 loss of function plants showed slow growth, with serrated and scrunched leaves, and an abnormal venation pattern via rRNA processing [4]. A transcriptome analysis in Arabidopsis revealed that several PUF members, in particular APUM9 and APUM11, showed higher transcript levels in reduced dormancy 5 mutant during seed imbibition. This study indicated that PUF proteins might also be involved in seed dormancy in plants [63]. Some studies showed that APUM-1 to APUM-6 may be involved in Arabidopsis growth and development in the early stage through binging to the RNA of their target genes such as *CLAVATA-1*, *WUSCHEL*, *FASCIATA-2*, and *PINHEAD/ZWILLE,* which are involved in the regulation of meristem growth and stem cell maintenance [42,83]. PUF protein APUM24 was also recently described as expressed in tissues undergoing rapid proliferation and cell division [65]. Moreover, APUM24 is required for timely removal of rRNA byproducts for rapid cell division and early embryogenesis in Arabidopsis. APUM24 loss of function plants displayed defects in cell patterning. 

## 4. PUF Proteins Control Post-Transcriptional Processes through Different Mechanisms

**Figure 2:**
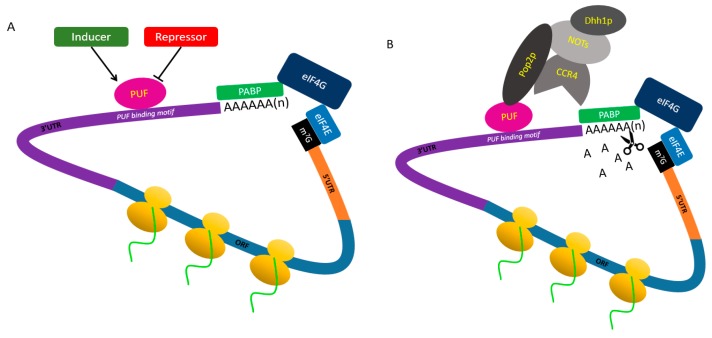
The common model of PUF protein influences mRNA stability. (**A**) Some genes regulate the expression or binding efficiency of PUF proteins in nucleus and/or in cytoplasm; (**B**) The PUF protein can bind to the PUF binding motif, which is located in the 3′UTR of target gene, and recruit the CCR4-POP2-NOT complex through interacting with Pop2 (the subunit of CCR4-POP2-NOT complex). The CCR4-POP2-NOT complex acts as a deadenylase in cell. It can affect the mRNA stability by reducing the length of poly(A) tail. Meanwhile, Dhh1p (DExD/H-box ATP-dependent RNA helicase), which can interact with CCR4-POP2-NOT complex, acts on the cap to activate decapping and inhibit translation [60].

PUF proteins exert their post-transcriptional action through various mechanisms such as activation of mRNA translation, repression of mRNA translation, and localization of mRNA [58,84,85]. One PUF repression mechanism probably correlates with shortening of the poly(A) tail of target mRNAs though deadenylation and repression awaits further research [1] (Figure 2). In yeast, PUF6p inhibits the initiation of *ASH1* mRNA translation via interactions with Fun12p during its transport; this repression can be relieved by CK2 phosphorylation in the N-terminal region of PUF6p when the mRNA reaches the bud tip [86]. PUF6p can also form a protein–RNA complex with She2p and repress translation by interacting with translation initiation factors and preventing ribosome transit^12^. Mpt5p, a yeast PUF protein, regulates *HO* mRNA and triggers shortening its poly(A) tail. A yeast PUF protein physically binds Pop2p (a component of the Ccr4p–Pop2p–Not deadenylase complex) required for PUF repression activity. Simultaneously, the PUF protein recruits deadenylase Ccr4p and Dcp1p and Dhh1p, which are involved in mRNA regulation. The PUF-Pop2p interaction is conserved in yeast, worms, and humans [60]. 

In *Caenorhabditis elegans*, FBF regulates the activation of *gld-1* (*defective in germline development-1*). A possible mechanism of that regulation is linked to cytoplasmic polyadenylation, i.e., extension of the mRNA poly(A) tail by cytoplasmic poly(A) polymerase [13]. FBF interacts with gld-1 mRNA and with the cytoplasmic polyadenylase, which it recruits [87]. However, it is also involved in another mechanism. In fact, FBF can bind the 3′UTR of EGL-4, a cGMP-dependent protein kinase, and may localize translation near the sensory cilia and cell body. Furthermore, the photoconvertible stony coral protein Kaede was used as a reporter gene in that experiment. The cell biology analysis showed that the subcellular distribution of newly synthesized Kaede dramatically changed in the *fbf-1* mutant. This result suggests that the binding of FBF may direct the subcellular localization of EGL-4 translation and enhance its translation [88]. In humans, Nop9 is a PUF-like protein. It recognizes sequences and structural features of 20S pre-rRNA near Nob1, the cleavage site of the nuclease and thus reduces Nob1 cleavage efficiency [78]. Nob1 cleavage is the final processing step in the production of mature 18S small subunit ribosomal RNA.

## 5. Conclusions

Post-transcriptional regulation is an essential component of gene expression regulation. Numerous studies conducted over several decades have unveiled and characterized many factors involved in post-transcriptional regulation, such as micro-RNAs, poly(A)-binding proteins (PABPs), small nuclear RNAs (snRNAs), or RNA-binding proteins (RBPs). PUF family RNA-binding proteins are determining post-transcriptional regulators present throughout eukaryotes. PUF proteins influence many aspects of different metabolic pathways, and the expression of PUF genes is regulated by many endogenous signals [25,48,89,90]. This article provides an overview of PUF proteins, i.e., their RNA targets, biological functions, and regulation mechanisms. These findings may lead us to discover more information and functions about plant PUF proteins, as current knowledge about the regulation of PUF gene expression and their role in plant biology is scarce. Most studies on plant PUF proteins have only focused on *Arabidopsis thaliana*, in which 26 PUF family members have been reported [7,42]. The relevance of PUF proteins in specific plant developmental processes such as branching, rhizogenesis, flowering, that are well known to be finely and flexibly controlled by endogenous and exogenous stimuli, still remains to be investigated. For example, the 3′UTR of some branching related genes in Arabidopsis, such as More Axillary Branches 2 (AtMAX2) and Smax1-like 6 (SMXL6), contained the putative binding sites of PUF protein. In addition, the putative PUF binding sites also were found in the 3′UTR of Flower Locus T (FT) and Terminal Flower 1 (TFL1), which are related to flowering in Arabidopsis. The 3′UTR of Retarded Root Growth (RRG), a rhizogenesis related gene, harbors many putative PUF binding sites. Therefore, some plant developmental processes may be controlled by PUF protein at the post-transcriptional level. The detail mechanism of these developmental processes needs to be studied deeply in the future.

## Figures and Tables

**Table 1:** Biological functions, binding motifs, and target mRNA of some of the most described PUF proteins in different living organisms.

Organisms	PUF Family Member	Target mRNA	Binding Motif	Biological Function	References
*Caenorhabditis elegans*	FBF	*Gld-1, Fem3*	5′-UGUGCCAUA-3′, 5′-UGUGUCAUU-3′	Maintenance of stem cell proliferation; the hermaphroditic switch between spermatogenesis and oogenesis; adaptation in the AWC chemosensory neuron.	[29,48,49]
	PUF-5	*HIS3* (a reporter gene), *obr-3*, *cpi-2*, *srm-6*, *fog-1*, *srz-10*, *C17H11*	5′-CYCUGUAYYYUGU-3′	Oocyte maturation; nuclear enlargement; yolk uptake; early embryogenesis	[49,50]
	PUF-6	*HIS3* (a reporter gene)	5′-CYCUGUAYYYUGU-3′	Primordial germ cell development	[49,50]
	PUF-7	*HIS3* (a reporter gene)	5′-CYCUGUAYYYUGU-3′	Primordial germ cell development	[49,50]
	PUF-8	Unknown	Unknown	Hermaphrodite spermoocyte switch; Germ-Line Proliferation	[49,51,52]
	PUF-9	Unknown	Unknown	Differentiation of epidermal stem cells at the larval-to-adult transition	[49,53]
*Cryptococcus neoformans*	PUM1	*Znf2*	5′-UGUACAUA-3′	Hyphal morphogenesis of sexual development	[41]
*Drosophila melanogaster*	PUMILIO	*hbNRE*; *hunchback*; *cyclin B*; *eIF4E*; *Bicoid*; *para*	Nanos response element	Anterior patterning system; mitotic arrest of primordial germ cells; maintenance of germline stem cells; primordial follicle pool; gonadogenesis; oogenesis; neuronal function; sodium current in motoneurons	[15,21,40,54,55,56,57,58]
*Saccharomyces cerevisiae*	MPT5	*HO*	Nanos response element	Mating-type switching; Lifespan	[23,59]
	PUF4	*HO*	Nanos response element	Lifespan	[1,59,60]
	PUF3	*COX17*	5′-UGUAUAUAU-3′	Mitochondrial biogenesis and motility; thermotolerance; hyperosmotic stress resistance	[32,59,61]
	PUF2	Unknown	5′-UAAUAAUUW-3′	Binds mRNAs encoding membrane-associated proteins	[59,62]
	PUF1/JSN1	Unknown	Unknown	A high copy suppressor of certain tubulin mutations	[59,63]
	PUF6	*ASH1*	5′-UUGU-3′ motif	Mating-type switching; protein/peptide accumulation	[12,59]
*Xenopus*	XPum2	*Xenopus cyclin B1*	5′-UGUAAAUA-3′	Oocyte maturation	[22,25,64]
*Arabidopsis thaliana*	APUM1-6	*FASCIATA-2*, *CLAVATA-1* and *ZWILLE⁄PINHEAD*	5′UGUANAUA	shoot meristem organization, stem cell maintenance and maintenance of cellular organization of apical meristems	[42]
	APUM5	CMV tripartite RNA 3′UTR regions	5′-UGUAAUA-3′; 5′-UGUAGUA-3′; 5′-UGUACAUAAUA-3′	Defensive repressor of *Cucumber mosaic virus* (CMV) infection	[65]
	APUM5	*RAB18 COR15 RD22 DREB2A*	5′-UGUA-3′	Abiotic stress response	[66]
	APUM9 and APUM11	Unknown	Unknown	Seed dormancy	[67]
	APUM23	Unknown	Unknown	Leaf development and organ polarity; Processing and/or degradation of 35S pre-rRNA and rRNA maturation by-products	[4,68]
	APUM24	7S pre-rRNA; *ITS2*	Unknown	rRNA processing and early embryogenesis	[69]

## References

[1] Wickens M., Bernstein D.S., Kimble J., Parker R. (2002). A PUF family portrait: 3′ UTR regulation as a way of life. Trends Genet..

[2] Glisovic T., Bachorik J.L., Yong J., Dreyfuss G. (2008). RNA-binding proteins and post-transcriptional gene regulation. FEBS Lett..

[3] Keene J.D. (2007). RNA regulons: Coordination of post-transcriptional events. Nat. Rev. Genet..

[4] Abbasi N., Kim H.B., Park N.I., Kim H.S., Kim Y.K., Park Y.I., Choi S.B. (2010). APUM23, a nucleolar PUF domain protein, is involved in pre-ribosomal RNA processing and normal growth patterning in Arabidopsis. Plant J..

[5] Ray D., Kazan H., Cook K.B., Weirauch M.T., Najafabadi H.S., Li X., Gueroussov S., Albu M., Zheng H., Yang A. (2013). A compendium of RNA-binding motifs for decoding gene regulation. Nature.

[6] Wu Z., Zhu D., Lin X., Miao J., Gu L., Deng X., Yang Q., Sun K., Zhu D., Cao X. (2016). RNA-binding proteins At RZ-1B and At RZ-1C play a critical role in regulation of pre-mRNA splicing and gene expression during Arabidopsis development. Plant Cell.

[7] Tam P.P., Barrette-Ng I.H., Simon D.M., Tam M.W., Ang A.L., Muench D.G. (2010). The PUF family of RNA-binding proteins in plants: Phylogeny, structural modeling, activity and subcellular localization. BMC Plant Biol..

[8] Friend K., Campbell Z.T., Cooke A., Kroll-Conner P., Wickens M.P., Kimble J. (2012). A conserved PUF–Ago–eEF1A complex attenuates translation elongation. Nat. Struct. Mol. Biol..

[9] Van Etten J., Schagat T.L., Hrit J., Weidmann C.A., Brumbaugh J., Coon J.J., Goldstrohm A.C. (2012). Human Pumilio proteins recruit multiple deadenylases to efficiently repress messenger RNAs. J. Biol. Chem..

[10] Miles W.O., Tschöp K., Herr A., Ji J.Y., Dyson N.J. (2012). Pumilio facilitates miRNA regulation of the E2F3 oncogene. Genes Dev..

[11] Lee S., Kopp F., Chang T.C., Sataluri A., Chen B., Sivakumar S., Yu H., Xie Y., Mendell J.T. (2016). Noncoding RNA NORAD regulates genomic stability by sequestering Pumilio proteins. Cell.

[12] Gu W., Deng Y., Zenklusen D., Singer R.H. (2004). A new yeast PUF family protein, PUF6p, represses ASH1 mRNA translation and is required for its localization. Genes Dev..

[13] Suh N., Crittenden S.L., Goldstrohm A., Hook B., Thompson B., Wickens M., Kimble J. (2009). FBF and its dual control of gld-1 expression in the *Caenorhabditis elegans* germline. Genetics.

[14] Zhang B., Gallegos M., Puoti A., Durkin E., Fields S., Kimble J., Wickens M.P. (1997). A conserved RNA-binding protein that regulates sexual fates in the *Caenorhabditis elegans* hermaphrodite germ line. Nature.

[15] Barker D.D., Wang C., Moore J., Dickinson L.K., Lehmann R. (1992). Pumilio is essential for function but not for distribution of the Drosophila abdominal determinant Nanos. Genes Dev..

[16] Zamore P.D., Williamson J.R., Lehmann R. (1997). The Pumilio protein binds RNA through a conserved domain that defines a new class of RNA-binding proteins. RNA.

[17] Wang X., Zamore P.D., Hall T.M.T. (2001). Crystal structure of a Pumilio homology domain. Mol. Cell.

[18] Hall T.M.T. (2016). De-coding and re-coding RNA recognition by PUF and PPR repeat proteins. Curr. Opin. Struct. Biol..

[19] Wang X., McLachlan J., Zamore P.D., Hall T.M.T. (2002). Modular recognition of RNA by a human pumilio-homology domain. Cell.

[20] Ahringer J., Kimble J. (1991). Control of the sperm-oocyte switch in *Caenorhabditis elegans* hermaphrodites by the fem-3 3′ untranslated region. Nature.

[21] Sonoda J., Wharton R.P. (1999). Recruitment of Nanos to hunchback mRNA by Pumilio. Genes Dev..

[22] Nakahata S., Katsu Y., Mita K., Inoue K., Nagahama Y., Yamashita M. (2001). Biochemical identification of Xenopus Pumilio as a sequence-specific cyclin B1 mRNA-binding protein that physically interacts with a Nanos homolog, Xcat-2, and a cytoplasmic polyadenylation element-binding protein. J. Biol. Chem..

[23] Tadauchi T., Matsumoto K., Herskowitz I., Irie K. (2001). Post-transcriptional regulation through the HO 3′ UTR by Mpt5, a yeast homolog of Pumilio and FBF. EMBO J..

[24] Crittenden S.L., Bernstein D.S., Bachorik J.L., Thompson B.E., Gallegos M., Petcherski A.G., Moulder G., Barstead R., Wickens M., Kimble J. (2002). A conserved RNA-binding protein controls germline stem cells in *Caenorhabditis elegans*. Nature.

[25] Spassov D., Jurecic R. (2003). The PUF Family of RNA-binding Proteins: Does Evolutionarily Conserved Structure Equal Conserved Function?. IUBMB Life.

[26] Galgano A., Forrer M., Jaskiewicz L., Kanitz A., Zavolan M., Gerber A.P. (2008). Comparative analysis of mRNA targets for human PUF-family proteins suggests extensive interaction with the miRNA regulatory system. PLoS ONE.

[27] Zamore P.D., Bartel D.P., Lehmann R., Williamson J.R. (1999). The PUMILIO-RNA Interaction: A single RNA-binding domain monomer recognizes a bipartite target sequence. Biochemistry.

[28] Gupta Y.K., Lee T.H., Edwards T.A., Escalante C.R., Kadyrova L.Y., Wharton R.P., Aggarwal A.K. (2009). Co-occupancy of two Pumilio molecules on a single hunchback NRE. RNA.

[29] Bernstein D., Hook B., Hajarnavis A., Opperman L., Wickens M. (2005). Binding specificity and mRNA targets of a *Caenorhabditis elegans* PUF protein, FBF-1. RNA.

[30] Qiu C., Kershner A., Wang Y., Holley C.P., Wilinski D., Keles S., Kimble J., Wickens M., Hall T.M. (2012). Divergence of Pumilio/fem-3 mRNA binding factor (PUF) protein specificity through variations in an RNA-binding pocket. J. Biol. Chem..

[31] Valley C.T., Porter D.F., Qiu C., Campbell Z.T., Hall T.M.T., Wickens M. (2012). Patterns and plasticity in RNA-protein interactions enable recruitment of multiple proteins through a single site. Proc. Natl. Acad. Sci. USA.

[32] García-Rodríguez L.J., Gay A.C., Pon L.A. (2007). PUF3p, a Pumilio family RNA binding protein, localizes to mitochondria and regulates mitochondrial biogenesis and motility in budding yeast. J. Cell Biol..

[33] Gerber A.P., Herschlag D., Brown P.O. (2004). Extensive association of functionally and cytotopically related mRNAs with Puf family RNA-binding proteins in yeast. PLoS Biol..

[34] Porter D.F., Koh Y.Y., VanVeller B., Raines R.T., Wickens M. (2015). Target selection by natural and redesigned PUF proteins. Proc. Natl. Acad. Sci. USA.

[35] White E.K., Moore-Jarrett T., Ruley H.E. (2001). PUM2, a novel murine PUF protein, and its consensus RNA-binding site. RNA.

[36] Lu G., Dolgner S.J., Hall T.M.T. (2009). Understanding and engineering RNA sequence specificity of PUF proteins. Curr. Opin. Struct. Biol..

[37] Cheong C.G., Hall T.M.T. (2006). Engineering RNA sequence specificity of Pumilio repeats. Proc. Natl. Acad. Sci. USA.

[38] Gupta Y.K., Nair D.T., Wharton R.P., Aggarwal A.K. (2008). Structures of human Pumilio with noncognate RNAs reveal molecular mechanisms for binding promiscuity. Structure.

[39] Miller M.T., Higgin J.J., Hall T.M.T. (2008). Basis of altered RNA-binding specificity by PUF proteins revealed by crystal structures of yeast Puf4p. Nat. Struct. Mol. Biol..

[40] Muraro N.I., Weston A.J., Gerber A.P., Luschnig S., Moffat K.G., Baines R.A. (2008). Pumilio binds para mRNA and requires Nanos and Brat to regulate sodium current in Drosophila motoneurons. J. Neurosci..

[41] Kaur J.N., Panepinto J.C. (2016). Morphotype-specific effector functions of Cryptococcus neoformans PUM1. Sci. Rep..

[42] Francischini C.W., Quaggio R.B. (2009). Molecular characterization of Arabidopsis thaliana PUF proteins–binding specificity and target candidates. FEBS J..

[43] Zhang C., Muench D.G. (2015). A nucleolar PUF RNA-binding protein with specificity for a unique RNA sequence. J. Biol. Chem..

[44] Biasini M., Bienert S., Waterhouse A., Arnold K., Studer G., Schmidt T., Kiefer F., Cassarino T.G., Bertoni M., Bordoli L. (2014). SWISS-MODEL: Modelling protein tertiary and quaternary structure using evolutionary information. Nucleic Acids Res..

[45] Kiefer F., Arnold K., Künzli M., Bordoli L., Schwede T. (2009). The SWISS-MODEL Repository and associated resources. Nucleic Acids Res..

[46] Arnold K., Bordoli L., Kopp J., Schwede T. (2006). The SWISS-MODEL Workspace: A web-based environment for protein structure homology modelling. Bioinformatics.

[47] Guex N., Peitsch M.C., Schwede T. (2009). Automated comparative protein structure modeling with SWISS-MODEL and Swiss-PdbViewer: A historical perspective. Electrophoresis.

[48] Kraemer B., Crittenden S., Gallegos M., Moulder G., Barstead R., Kimble J., Wickens M. (1999). NANOS-3 and FBF proteins physically interact to control the sperm–oocyte switch in *Caenorhabditis elegans*. Curr. Biol..

[49] Stein L., Sternberg P., Durbin R., Thierry-Mieg J., Spieth J. (2001). WormBase: Network access to the genome and biology of *Caenorhabditis elegans*. Nucleic Acids Res..

[50] Stumpf C.R., Kimble J., Wickens M. (2008). A *Caenorhabditis elegans* PUF protein family with distinct RNA binding specificity. RNA.

[51] Bachorik J.L., Kimble J. (2005). Redundant control of the *Caenorhabditis elegans* sperm/oocyte switch by PUF-8 and FBF-1, two distinct PUF RNA-binding proteins. Proc. Natl. Acad. Sci. USA.

[52] Ariz M., Mainpal R., Subramaniam K. (2009). *Caenorhabditis elegans* RNA-binding proteins PUF-8 and MEX-3 function redundantly to promote germline stem cell mitosis. Dev. Biol..

[53] Nolde M.J., Saka N., Reinert K.L., Slack F.J. (2007). The *Caenorhabditis elegans* pumilio homolog, puf-9, is required for the 3′ UTR-mediated repression of the let-7 microRNA target gene, hbl-1. Dev. Biol..

[54] Forbes A., Lehmann R. (1998). Nanos and Pumilio have critical roles in the development and function of Drosophila germline stem cells. Development.

[55] Asaoka-Taguchi M., Yamada M., Nakamura A., Hanyu K., Kobayashi S. (1999). Maternal Pumilio acts together with Nanos in germline development in Drosophila embryos. Nat. Cell Biol..

[56] Parisi M., Lin H. (1999). The Drosophila pumilio gene encodes two functional protein isoforms that play multiple roles in germline development, gonadogenesis, oogenesis and embryogenesis. Genetics.

[57] Mak W., Fang C., Holden T., Dratver M.B., Lin H. (2016). An important role of Pumilio 1 in regulating the development of the mammalian female germline 1. Biol. Reprod..

[58] Quenault T., Lithgow T., Traven A. (2011). PUF proteins: Repression, activation and mRNA localization. Trends Cell Biol..

[59] Cherry J.M., Hong E.L., Amundsen C., Balakrishnan R., Binkley G., Chan E.T., Christie K.R., Costanzo M.C., Dwight S.S., Engel S.R. (2011). Saccharomyces genome database: The genomics resource of budding yeast. Nucleic Acids Res..

[60] Goldstrohm A.C., Hook B.A., Seay D.J., Wickens M. (2006). PUF proteins bind Pop2p to regulate messenger RNAs. Nat. Struct. Mol. Biol..

[61] Olivas W., Parker R. (2000). The Puf3 protein is a transcript-specific regulator of mRNA degradation in yeast. EMBO J..

[62] Yosefzon Y., Koh Y.Y., Chritton J.J., Lande A., Leibovich L., Barziv L., Petzold C., Yakhini Z., Mandel-Gutfreund Y., Wickens M. (2011). Divergent RNA binding specificity of yeast Puf2p. RNA.

[63] Machin N.A., Lee J.M., Barnes G. (1995). Microtubule stability in budding yeast: Characterization and dosage suppression of a benomyl-dependent tubulin mutant. Mol. Biol. Cell.

[64] Jalal Kiani S., Taheri T., Rafati S., Samimi-Rad K. (2017). PUF proteins: Cellular functions and potential applications. Curr. Protein Pept. Sci..

[65] Huh S.U., Kim M.J., Paek K.H. (2013). Arabidopsis Pumilio protein APUM5 suppresses cucumber mosaic virus infection via direct binding of viral RNAs. Proc. Natl. Acad. Sci. USA.

[66] Huh S.U., Paek K.H. (2014). APUM5, encoding a Pumilio RNA binding protein, negatively regulates abiotic stress responsive gene expression. BMC Plant Biol..

[67] Xiang Y., Nakabayashi K., Ding J., He F., Bentsink L., Soppe W.J. (2014). Reduced Dormancy5 encodes a protein phosphatase 2C that is required for seed dormancy in Arabidopsis. Plant Cell.

[68] Huang T., Kerstetter R.A., Irish V.F. (2014). APUM23, a PUF family protein, functions in leaf development and organ polarity in Arabidopsis. J. Exp. Bot..

[69] Shanmugam T., Abbasi N., Kim H.S., Kim H.B., Park N.I., Park G.T., Oh S.A., Park S.K., Muench D.G., Choi Y. (2017). An arabidopsis divergent Pumilio protein, APUM24, Is essential for embryogenesis and required for faithful pre-rRNA processing. Plant J..

[70] Weidmann C.A., Goldstrohm A.C. (2012). Drosophila Pumilio protein contains multiple autonomous repression domains that regulate mRNAs independently of Nanos and brain tumor. Mol. Cell. Biol..

[71] Racher H., Hansen D. (2012). PUF-8, a Pumilio homolog, inhibits the proliferative fate in the *Caenorhabditis elegans* germline. G3 Genes Genomes Genet..

[72] Hubstenberger A., Cameron C., Shtofman R., Gutman S., Evans T.C. (2012). A network of PUF proteins and Ras signaling promote mRNA repression and oogenesis in *Caenorhabditis elegans*. Dev. Biol..

[73] Salvetti A., Rossi L., Lena A., Batistoni R., Deri P., Rainaldi G., Locci M.T., Evangelista M., Gremigni V. (2005). DjPum, a homologue of Drosophila Pumilio, is essential to planarian stem cell maintenance. Development.

[74] Goldstrohm A.C., Seay D.J., Hook B.A., Wickens M. (2007). PUF protein-mediated deadenylation is catalyzed by Ccr4p. J. Biol. Chem..

[75] Herskowitz I. (1988). Life cycle of the budding yeast *Saccharomyces cerevisiae*. Microbiol. Rev..

[76] Zhu D., Stumpf C.R., Krahn J.M., Wickens M., Hall T.M.T. (2009). A 5′ cytosine binding pocket in Puf3p specifies regulation of mitochondrial mRNAs. Proc. Natl. Acad. Sci. USA.

[77] Wilinski D., Qiu C., Lapointe C.P., Nevil M., Campbell Z.T., Hall T.M.T., Wickens M. (2015). RNA regulatory networks diversified through curvature of the PUF protein scaffold. Nat. Commun..

[78] Zhang J., McCann K.L., Qiu C., Gonzalez L.E., Baserga S.J., Hall T.M.T. (2016). Nop9 is a PUF-like protein that prevents premature cleavage to correctly process pre-18S rRNA. Nat. Commun..

[79] Stewart M.S., Krause S.A., McGhie J., Gray J.V. (2007). Mpt5p, a stress tolerance-and lifespan-promoting PUF protein in *Saccharomyces cerevisiae*, acts upstream of the cell wall integrity pathway. Eukaryot. Cell.

[80] Jiang L., Ye W., Situ J., Chen Y., Yang X., Kong G., Liu Y., Tinashe R.J., Xi P., Wang Y. (2017). A PUF RNA-binding protein encoding gene *PlM90* regulates the sexual and asexual life stages of the litchi downy blight pathogen *Peronophythora litchii*. Fungal Genet. Biol..

[81] Shrestha S., Li X., Ning G., Miao J., Cui L. (2016). The RNA-binding protein Puf1 functions in the maintenance of gametocytes in *Plasmodium falciparum*. J. Cell Sci..

[82] Narita R., Takahasi K., Murakami E., Hirano E., Yamamoto S.P., Yoneyama M., Kato H., Fujita T. (2014). A novel function of human Pumilio proteins in cytoplasmic sensing of viral infection. PLoS Pathog..

[83] Reichel M., Liao Y., Rettel M., Ragan C., Evers M., Alleaume A.M., Horos R., Hentze M.W., Preiss T., Millar A.A. (2016). In planta determination of the mRNA-binding proteome of Arabidopsis etiolated seedlings. Plant Cell.

[84] Cooke A., Prigge A., Opperman L., Wickens M. (2011). Targeted translational regulation using the PUF protein family scaffold. Proc. Natl. Acad. Sci. USA.

[85] Abbasi N., Park Y.I., Choi S.B. (2011). Pumilio Puf domain RNA-binding proteins in Arabidopsis. Plant Signal. Behav..

[86] Deng Y., Singer R.H., Gu W. (2008). Translation of ASH1 mRNA is repressed by PUF6p–Fun12p/eIF5B interaction and released by CK2 phosphorylation. Genes Dev..

[87] Luitjens C., Gallegos M., Kraemer B., Kimble J., Wickens M. (2000). CPEB proteins control two key steps in spermatogenesis in *C. elegans*. Genes Dev..

[88] Kaye J.A., Rose N.C., Goldsworthy B., Goga A., Noelle D.L. (2009). A 3′ UTR pumilio-binding element directs translational activation in olfactory sensory neurons. Neuron.

[89] Lee M.H., Hook B., Pan G., Kershner A.M., Merritt C., Seydoux G., Thomson J.A., Wickens M., Kimble J. (2007). Conserved regulation of MAP kinase expression by PUF RNA-binding proteins. PLoS Genet..

[90] Moore F.L., Jaruzelska J., Fox M.S., Urano J., Firpo M.T., Turek P.J., Dorfman D.M., Pera R.A. (2003). Human Pumilio-2 is expressed in embryonic stem cells and germ cells and interacts with DAZ (Deleted in AZoospermia) and DAZ-like proteins. Proc. Natl. Acad. Sci. USA.

